# Bacteriocin-Like Inhibitory Substances from Probiotics as Therapeutic Agents for *Candida* Vulvovaginitis

**DOI:** 10.3390/antibiotics10030306

**Published:** 2021-03-17

**Authors:** Enas Mamdouh Hefzy, Mahmoud A. F. Khalil, Amal A. Ibrahim Amin, Hossam M. Ashour, Yasser Fathy Abdelaliem

**Affiliations:** 1 Department of Medical Microbiology and Immunology, Faculty of Medicine, Fayoum University, Fayoum 63514, Egypt; emh01@fayoum.edu.eg (E.M.H.); aai02@fayoum.edu.eg (A.A.I.A.); 2 Department of Microbiology and Immunology, Faculty of Pharmacy, Fayoum University, Fayoum 63514, Egypt; maf04@fayoum.edu.eg; 3 Department of Integrative Biology, College of Arts and Sciences, University of South Florida, St. Petersburg, FL 33701, USA; 4 Department of Microbiology and Immunology, Faculty of Pharmacy, Cairo University, Cairo 11562, Egypt; 5 Department of Agricultural Microbiology, Faculty of Agriculture, Fayoum University, Fayoum 63514, Egypt; yfa00@fayoum.edu.eg

**Keywords:** bacteriocins, biofilm, *Galleria mellonella*, vulvovaginitis

## Abstract

Probiotics can potentially prevent and treat diseases. We examined the inhibitory activity of bacteriocin-like inhibitory substances (BLISs) from potentially probiotic lactobacilli and streptococci on *Candida albicans* and non-*Candida albicans* clinical isolates from women with vulvovaginitis. Using agar well diffusion assays, BLISs inhibited both *Candida albicans* and non-*Candida albicans* isolates. The BLIS from *L. pentosus* isolates had the highest anti-*Candida* activity (33/45; 73.3%), followed by BLISs from isolates of *L. paracasei* subsp. *paracasei* (31/45; 68.9%), *L. rhamnosus I* (30/45; 66.7%)*, L. delbrueckii* subsp. *lactis I* (30/45; 66.7%), and *S. uberis II* (30/45; 66.7%). Upon characterization according to the retained activity under variable physical and chemical conditions, the BLISs showed stability against heat, pH, and surfactants, but were protease-sensitive, which suggests a proteinaceous nature of the active substances. Using crystal violet assays, the BLISs reduced the *Candida* biofilm biomass significantly as compared to a control group that lacked BLISs. In vivo testing of the antagonistic activity was performed using the *Galleria mellonella* (*G. mellonella*) larvae model. BLISs significantly improved survival in *G. mellonella* larvae treated with *Candida* isolates on the first, second, and seventh days, as compared to larvae inoculated with *Candida* only (*p* < 0.01). The results show that BLISs can be used as biotherapeutic agents in vulvovaginal candidiasis.

## 1. Introduction

Vulvovaginal candidiasis (VVC) is a common condition that is typically mitigated by over-the-counter topical or systemic antifungal drugs. About 75% of women experience one or more VVC attacks, whereas 10% experience recurrent VVC attacks [1]. Pathogenic *Candida* species have developed resistance to many antifungal agents [2]. This highlights the need to develop alternative antimycotic agents to control pathogenic *Candida* infections [3]. In addition to resistance issues, many currently available antifungal drugs have narrow spectra and cause side effects [4].

*Lactobacilli* are dominant in the vagina of the vast majority of healthy premenopausal women [5]. As part of the microbiota, lactobacilli can prevent vulvovaginal infections [6]. The antifungal activities are not always explained by hydrogen peroxide, a typical antimicrobial factor in lactobacilli [7]. The potent antifungal activity of probiotic strains of *Lactobacillus* can be partially explained by the low pH and the production of organic acids [8]. In addition, lactic acid bacteria such as *Enterococci* and *Streptococci* can produce antimicrobial peptides such as bacteriocins [9]. As an example, *Streptococcus uberis*, the main causative agent of bovine mastitis, produces a lantibiotic bacteriocin (nisin U) [10]. In addition, *Lactobacillus plantarum* and *Lactobacillus curvatus* are able to synthesize bacteriocins [11].

Bacteriocins are low-molecular-mass peptides synthesized by bacterial ribosomes and released extracellularly to kill or inhibit other bacterial strains [12]. Bacteriocins have three major classes based on structure, physiochemical and molecular characteristics, and antimicrobial activity [13]. Bacteriocin-like inhibitory substances (BLISs) are uncharacterized substances with bacteriocin-like inhibitory activity [14].

A vital virulence factor of *Candida albicans* (*C. albicans*) that has been associated with the development and recurrence of vaginal candidiasis is biofilm formation [15]. The biofilm structure consists of microbial communities entrenched in an extracellular matrix [16]. Biofilms are extremely difficult to eliminate due to the physical exclusion of antimicrobial agents, induction of efflux pump activity, and the presence of persister cells that are the basis for chronic infections. Due to the importance of eradicating biofilms, new treatment strategies are being tested, such as the application of probiotics into the oral cavity in order to control oral biofilms [17]. *C. albicans* biofilms can also be inhibited by probiotic lactobacilli that can produce bacteriocins, suppress adhesion receptors, and modulate the immune system [18].

As a model organism for the study of bacterial and fungal infections, *Galleria mellonella* (*G. mellonella*), also known as the greater wax moth, has many advantages. The 20 mm long larvae are cheap, easy to handle, and can be propagated at 37 °C. The model can be used to study *C. albicans* virulence factors including biofilm development and to assess potential antifungal agents [19]. The model can also be used to assess the effect of probiotics on microbial pathogens [20].

Here, we used the *G. mellonella* in vivo model to assess the antagonistic and antibiofilm activities of BLISs produced by lactobacilli and streptococci isolated from food sources against *Candida* strains isolated from women with vulvovaginitis. The study assesses whether BLISs from potential probiotics can act as therapeutic agents for *Candida* vulvovaginitis.

## 2. Materials and Methods

### 2.1. Isolation of Microorganisms

#### 2.1.1. Isolation and Identification of Potential Probiotics

Fifty potential probiotic isolates were obtained from samples of raw milk, fermented milk, yogurt, cheese, meat products, and mixed pickles. *Lactobacillus* isolation on de Man, Rogosa, Sharpe (MRS) agar (Oxoid, Basingstoke, UK), and *Streptococcus* isolation on M17 agar (Oxoid, Basingstoke, UK) were performed [21]. To identify the isolated bacteria, standard microbiological methods (phenotypic, morphological, and biochemical techniques) were used. All isolates were grown at 15 °C and 45 °C. Gram staining, catalase tests, and glucose fermentation tests were performed [22]. Carbohydrate fermentation patterns for the isolated lactobacilli and streptococci were determined using the API Rapid CH fermentation strips (Biomèrieux, Marcy l’Etoile, France) in CHL medium and the API 20 Strep kit (Biomèrieux, Marcy l’Etoile, France) respectively.

#### 2.1.2. Isolation and Identification of *Candida* Isolates

Vaginal swabs from females with vulvovaginitis were inoculated on Sabouraud dextrose agar (SDA) (Oxoid, Basingstoke, UK) and incubated for 24–48 h at 37 °C. Isolated colonies were identified using standard microbiological methods (colony characteristics, Gram staining, urea hydrolysis, and germ tube test) [23]. The creamy yeast-like colonies that were Gram-positive showed pseudohyphae under a light microscope, and negative results with the urea hydrolysis test were further examined for *Candida* species identification [23]. Carbohydrate assimilation was tested using API *Candida* (BioMèrieux, Marcy-l’Etoile, France). Forty-five different clinical isolates were retrieved, including twenty-five *C. albicans* isolates (CA 1-25) and 20 non-*C. albicans Candida* isolates (NCAC 1-20).

#### 2.1.3. Screening for Anti-*Candida* Activity

Agar well diffusion was used to assess the BLIS inhibitory potential against *Candida* isolates causing vulvovaginitis [24]. Potential probiotics showing anti-*Candida* activities against at least one of two indicator organisms: *C. albicans* (ATCC 90028) and *C. glabrata* (ATCC 90030), were selected for further processing after confirmation by API (as described in Section 2.1.1). BLISs were extracted from potential probiotics (Section 2.2) and subsequently examined for anti-*Candida* activity. BLISs with the strongest activities were further characterized (Section 2.3).

BLISs from thirteen potential probiotic isolates (13/50; 26%) showed anti-*Candida* activity against at least one indicator organism, which was determined by the residual activity after pH neutralization and hydrogen peroxide elimination. Isolates, as identified by conventional bacteriological methods and the API systems, included one *L. pentosus* isolate, two *L. plantarum* isolates, two *L. rhamnosus* isolates, one *L. delbrueckii* subsp. *bulgaricus* isolate, two *L. paracasei* subsp. *paracasei* isolates, two *L. delbrueckii* subsp. *Lactis* I and II isolates, one *S. agalactiae* isolate, and two *S. uberis* isolates (Table 1).

The activity of the thirteen potential probiotic isolates was assessed against the 45 clinical isolates. Twenty-five *C. albicans* (CA 1-25) and twenty non-*C. albicans* (NCAC 1-20) were cultured on Sabouraud Dextrose broth (SDB) (Oxoid, Basingstoke, UK) at 37 °C for 24 h. *C. albicans* (ATCC 90028) and *C. glabrata* (ATCC 90030) strains were used as indicator organisms. The NCAC group contained fourteen *Candida glabrata* (*C. glabrata*), two *Candida tropicalis (C. tropicalis*), two *Candida famata (C. famata*), and two *Candida kruesi* (*C. kruesi*).

### 2.2. Preparation of BLISs

Overnight cultures of the potential probiotic isolates cultivated on broth medium were diluted with fresh medium (inoculum size 1% *v*/*v*) at 32 °C for 18 h, and the concentration was adjusted to an optical density of 1.6 at 600 nm (~1 × 10^8^ cells/mL). MRS broth (Oxoid, Basingstoke, UK) was used for the cultivation of lactobacilli, and M17 broth (Oxoid, Basingstoke, UK) was used for the cultivation of streptococci. Cultures were centrifuged at 10,000× *g* for 15 min at 4 °C and the resulting supernatant was designated as a crude cell-free culture supernatant (CCFCS). To neutralize hydrogen peroxide, 1 mg/mL of bovine catalase (Sigma-Aldrich, St. Louis, MO, USA) was added. The pH of each CCFCS was adjusted to 6.5 with 1 mol/L NaOH (Sigma-Aldrich, St. Louis, MO, USA) [21]. The treated supernatant was designated a BLIS. The 10-fold concentration of the BLIS was obtained using a vacuum rotary evaporator at 40 °C, filtered through a sterile 0.2 μm syringe filter, and stored at −20 °C until further use.

### 2.3. Physicochemical Characterization of BLISs

The five BLISs with the most potent anti-*Candida* activity were selected for further characterization and assessment of the antimicrobial activity. The residual anti-*Candida* activity of treated BLISs under different conditions was determined by agar diffusion and compared with the positive control (untreated, 100% activity).

(1) Effect of heating: BLISs were incubated in a water bath at 60 °C, 80 °C, and 100 °C for 10, 30, 60 min and at 121 °C for 10, 15, and 20 min, and then cooled on ice.
(2) Effect of pH: Catalase-treated BLISs were adjusted to pH 3.0, 5.0, 7.0, and 10.0 by hydrochloric acid and sodium hydroxide, and allowed to stand at room temperature for 2 h.
(3) The sensitivity of BLISs to proteases (pepsin and trypsin), in addition to α-amylase (Sigma-Aldrich, St. Louis, MO, USA), was assessed (final concentration of 1 mg/mL). Samples with and without enzymes were incubated for 3 h at 30 °C.
(4) Effect of organic solvents: Chloroform, ethanol, and n-hexane at concentrations of 10, 15, 20, and 30% (*v*/*v*) were determined. Samples with and without solvents, as well as solvents only, were incubated at 30 °C for 1, 4, 6, and 24 h.
(5) The effect of surfactants (Tween-20 and Tween-80) at concentrations of 0.1%, 1%, 2%, and 5.0% (*v*/*v*) was determined. Surfactants were added to BLISs at a 0.1 mL concentration of surfactant/mL of bacteriocin solutions. Samples, with and without surfactants, as well as surfactants only, were incubated at 30 °C for 2 h.

### 2.4. Protein Purification and Separation from BLIS

The protein content of the cell-free BLIS was isolated by overnight precipitation with ammonium sulphate at a saturation level of 70% with slight agitation at 4 °C [25]. The anti-*Candida* activity of the ammonium sulfate-precipitated BLIS was determined by the agar well diffusion assay using indicator strains. The BLIS was purified before measuring its protein concentration in the supernatant [26]. The molecular mass of the partially purified BLIS was estimated by SDS-PAGE (sodium dodecyl sulfate-polyacrylamide gel electrophoresis) with 4% stacking and 10% polyacrylamide gel [27].

### 2.5. In Vitro Anti-biofilm Activity of BLIS

The antibiofilm activities of the BLISs from five potential probiotics with the strongest activities against *Candida*, as detected by agar well diffusion, were tested. BLISs from *L. pentosus, L. rhamnosus I, L. paracasei* subsp. *paracasei II, L. delbrueckii* subsp. *lactis I*, and *S. uberis II.* were freshly prepared, and viable counts were determined to verify their cell-free status before each experiment. They were then tested against biofilms formed by *C. albicans* ATCC 90028, *C. albicans* (CA 1), and *C. glabrata* (belongs to NCAC 1) clinical isolates. A standard inoculum of 1 × 10^6^ cells from the overnight culture of each fungal strain was used to form the biofilm. In each experiment, 40 µL per well of the tested BLISs was added to wells of sterile flat-bottomed polystyrene 96-well microtiter plates, followed by the addition of the tested *Candida* culture (160 µL) to a final volume of 200 µL/well [20].

For each experiment, two control groups were prepared: One that had wells with phosphate-buffered saline (PBS) only, and one that had wells with standardized BLISs (without *C. albicans*) from each of the five forementioned probiotics. After daily media change, plates were incubated for 48 h at 37 °C, with shaking at 75 rpm. After biofilm formation, biofilm biomasses with and without BLISs were quantified using crystal violet assays [28]. The absorbance was quantified at 540 nm. The reference strain *C. albicans* DAY185 was used as a positive control [29].

### 2.6. G. mellonella Survival Assay for BLIS Activity Against Candida Isolates (In Vivo Model)

The pathogenicity of *C. albicans* ATCC 90028, CA 1, and NCAC 1 clinical isolates in the presence or absence of cell-free BLISs was assessed using *G. mellonella* survival assays. Sixteen *G. mellonella* larvae in the final larval stage with similar masses (250–350 mg) and sizes were used [30]. Three control groups of noninfected larvae were included as controls: Group 1 was inoculated with PBS to assess for potential physical trauma; Group 2 was inoculated with MRS broth to evaluate for any toxicity on *G. mellonella* larvae; Group 3 was not injected (control for overall viability). A 5 µL inoculum of the standard *Candida* isolate suspension (10^5^ cells/mL) was injected into the hemolymph of each larva through the last left proleg, and 5 µL of each BLIS was injected into the last right proleg. For the groups infected with *Candida* isolates only, 5 µL of the microbial suspension was inoculated into the last left proleg and 5 µL of PBS was injected into the last right proleg. In these experiments, *Candida* isolates were inoculated 1 h before the inoculation of the BLISs. The larvae were incubated at 37 °C. When a larva displayed no movement in response to stimuli or showed dark discoloration of the cuticle, it was considered dead. Dead larvae were counted daily for 7 days [30].

### 2.7. Polymerase Chain Reaction (PCR) Amplification and Identification of Lactobacilli by Partial Sequencing of the 16S rRNA Gene

The genomic DNA of potential probiotics with the strongest in vitro anti-*Candida* activities was extracted and purified using DNeasy blood and tissue kits (Qiagen Inc., Hilden, Germany). The 16S rRNA gene was amplified using universal bacterial primers (518F/800R) [31]. PCR fragment analysis was done [32]. Sequences were compared, using BLAST, to those in the GenBank database.

### 2.8. Statistical Analysis

Data analysis was done using SPSS 16 (SPSS Inc., Chicago, IL, USA) and the chi-square test. Fisher’s exact test was used for two-by-two tables when the expected cell counts were less than 5. The *G. mellonella* survival curve was analyzed using the log-rank test. 

## 3. Results

### 3.1. Isolation of Active Lactobacilli and Streptococci and Screening for BLIS Antagonistic Activity

The BLISs of the thirteen probiotic isolates (with activity against one or more indicator organisms) were extracted and tested for anti-*Candida* activity against twenty-five *C. albicans* (CA 1-25) and twenty non-*C. albicans* (NCAC 1-20) (Table 1). The inhibitory activity against all *Candida* isolates ranged from 51.1% (23/45 isolates) for the BLIS from *Streptococcus uberis I* to 73.3% (33/45 isolates) for the BLIS from *L. pentosus*. The BLIS from *L. pentosus* showed a significantly higher antagonistic activity against NCAC isolates than against CA isolates (90% versus 60%, *p* = 0.024). BLISs from other potential probiotic isolates did not show a significant difference in antagonistic activity against NCAC isolates versus CA isolates (Table 1). The BLIS from *L. pentosus* isolates had the highest anti-*Candida* activity (33/45; 73.3%), followed by the BLIS from isolates of *L. paracasei* subsp. *paracasei* (31/45; 68.9%), *L. rhamnosus I* (30/45; 66.7%)*, L. delbrueckii* subsp. *lactis I* (30/45; 66.7%), and *S. uberis II* (30/45; 66.7%) (Table 1). The previous five BLISs with the most potent anti-*Candida* activity were selected for further characterization and assessment against *C. albicans* ATCC 90028 and *C. glabrata* ATCC 90030 indicator strains.

### 3.2. BLIS Characterization

The five BLISs with the highest antimicrobial activity were reassessed after exposure to heat, enzymes, pH, surfactants, and organic solvents. Variable degrees of stability of BLISs after treatment with these factors were reported (Table 2).

All BLISs retained their activity at 10% concentration of the three organic solvents: Chloroform, ethanol, and n-hexane, for 1, 4, and 6 h against both *C. albicans* and *C. glabrata.* With chloroform, all BLISs completely lost their activity at 30% concentrations at 1, 4, 6, and 24 h. With ethanol, only the BLISs from *L. paracasei* subsp. *paracasei II* retained ~50% of their activity against *C. albicans* at 30% concentration for 1 h. The BLIS from *L. pentosus* retained 75% of its activity against *C. glabrata* at 20% concentration of ethanol for 4 h.

BLISs were completely inactivated by pepsin and trypsin but not by α-amylase (Table 2). BLISs retained different levels of activity after treatment with surfactants. In some cases, an enhancement of activity (>100%) was observed (Table 3).

Partial purification of BLISs was accomplished by ammonium sulphate precipitation. Precipitates at lower ammonium sulphate saturation had lower anti-*Candida* activity. Maximal activity was observed at 70% ammonium sulphate saturation. Using SDS-PAGE followed by Coomassie blue staining, the purified BLISs showed protein bands with molecular weights ranging from 2.5 to 10.5 kDa.

### 3.3. Effect of BLIS on Candida Biofilm Formation In Vitro

*C. albicans* biofilm formation is a key mechanism for its growth and survival in the host. Using crystal violet assays, BLISs from *L. pentosus, L. paracasei* subsp. *paracasei II*, *L. rhamnosus I*, *L. delbrueckii* subsp. *lactis I*, and *S. uberis II* significantly reduced biofilm formation (expressed as relative absorbance) by *C. albicans* ATCC 90028, CA 1, and NCAC 1 clinical isolates. A control group that lacked BLISs failed to yield similar results. The BLIS from *L. pentosus* was most effective, whereas the BLIS from *S. uberis II* was least effective (Table 4).

### 3.4. BLIS Prolongs the Survival of Candida-Infected G. mellonella Larvae

The pathogenicity of *C. albicans* (ATCC 90028) and the clinical isolates CA 1 and NCAC 1, in the presence and absence of cell-free BLISs from *L. pentosus, L. paracasei* subsp. *paracasei II*, *L. rhamnosus I, L. delbrueckii* subsp. *lactis I*, and *S. uberis II*, was assessed using the *G. mellonella* killing assay.

Inoculation of the larvae with *Candida* isolates (in the absence of BLISs) killed 87.5% of the larvae within 24 h when CA 1 was injected, and 81.2% upon injection of NCAC 1 or *C. albicans* ATCC 90028 (Table 5).

**Figure 1:**
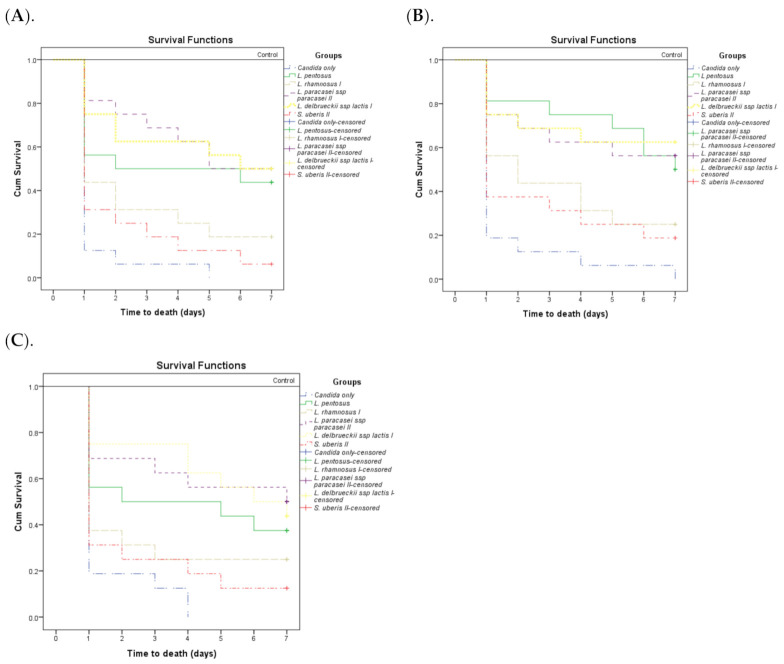
Pairwise comparison of the Kaplan–Meier cumulative (Cum) survival curves for *G. mellonella* larvae infected with *Candida* and injected with bacteriocin-like inhibitory substances (BLISs) from potential probiotic strains. The “*Candida* only” group represents *Candida* without the injection of BLISs. Control refers to the negative control injected with PBS only or with MRS broth only, or the noninjected control (0% larvae mortality in all three cases). (**A**) *C. albicans* clinical isolate (CA 1). (**B**) *C. glabrata* clinical isolate (NCAC 1). (**C**) *C. albicans* ATCC 90028.

Conversely, larvae treated with *Candida* isolates plus BLISs of *L. pentosus, L. delbrueckii* subsp. *lactis I*, and *L. paracasei* subsp. *paracasei II* (50 μg/larva) exhibited significantly improved survival on the first, second, and seventh days in comparison to larvae inoculated with *Candida* isolates alone (*p* < 0.01) (Table 5). The effect of the BLIS from *L. rhamnosus I* on the survival of *G. mellonella* larvae infected with *Candida* isolates on the first, second, and seventh days was less significant. Finally, the effect of the BLIS from *S. uberis II* was not statistically significant as compared to *Candida* isolates alone (Table 5 and Figure 1).

Control larvae (without *Candida* isolates or BLISs) exhibited 0% mortality on the first, second, and seventh days (Figure 1). These results showed that BLISs of potential probiotics protected *G. mellonella* larvae from *Candida*-induced mortality.

### 3.5. Identification of Lactobacilli by Partial Sequencing of the 16S rRNA Gene Sequences

PCR fragment analysis was done for the 16S rRNA gene sequences of potential probiotics with the best in vitro anti-*Candida* activity. After comparing sequences with those in the GenBank database, the 16S rRNA sequences were deposited in the GenBank database with the accession numbers LC406091, LC406092, LC406093, and LC406094 for *L. pentosus*, *L. rhamnosus*, *L. paracasei*, and *L. delbrueckii* subsp. *lactis*, respectively.

## 4. Discussion

In this study, we assessed the anti-*Candida* activity of BLISs from food-derived probiotic isolates. Notably, 26% of the isolates showed BLIS-mediated anti-*Candida* activity. In previous studies, the bacteriocinogenic strains ranged from 0.27% to 20% [33,34,35]. Variations in food samples and media used for the isolation of bacteriocinogenic lactobacilli and streptococci may have contributed to this difference. Results, in the present study, indicated that the BLIS from *L. pentosus* exhibited the highest anti-*Candida* activity. Pentocin TV35b, a BLIS isolated from *L. pentosus*, has also been reported to inhibit the growth of *C. albicans* [36].

Orally administered lactobacilli reduced vaginal colonization and infection by *Candida* [37]. Similarly, intravaginal capsules containing lactobacilli had anti-microbial effects against *C. albicans* and other pathogens [38,39]. The inhibitory activities of vaginal lactobacilli may be synergistically enhanced by the production of antimicrobials by the administered probiotics. The previous findings are consistent with our finding that lactobacilli can prevent VVC. Conversely, other studies have suggested that lactobacilli do not protect against VVC [7,40,41]. The ability of lactobacilli in the vaginal flora to produce potent antimicrobials can be impacted by a variety of dietary and environmental factors [42]. The previously reported potent antifungal activity of *Lactobacillus reuteri* RC-14 and *L. rhamnosus* GR-1 strains against *C. glabrata* causing VVC is consistent with the findings in this study [8].

The heat stability of BLISs reported in this study is consistent with reports of the heat stability of bacteriocin-like antimicrobial substances produced by bacteria in other studies [7,34,43,44]. The anti-*Candida* activity of BLISs was observed under acidic, neutral, and alkaline conditions (highest activity at pH 7), indicating that this activity was not due to acid production. Another report indicated that bacteriocin was stable in a pH range of 2 to 8 [45]. Other bacteriocins were reported to have high antibacterial activity at acidic pH [46]. Treatment of BLISs with organic solvents led to a reduced activity at high solvent concentrations. This may be due to the lipid moiety in BLISs [47]. Alcohol and chloroform inactivated bacteriocin from *L. plantarum*, but acetone, hexane, and alcohol (90%) did not inactivate bacteriocin from *Pediococcus pentosaceus* [46]. Organic solvents did not affect the inhibitory activities of some bacteriocins [47].

In line with previous studies, we observed BLIS inactivation by proteolytic (but not nonproteolytic) enzymes, which suggests a proteinaceous nature [33,43,48,49]. BLISs retained their activity after treatment with surfactants. In some cases, the antimicrobial activity was even enhanced (Table 3). This can be due to surfactants causing dispersion of bacteriocin complexes into active subunits with more lethal power [50]. The treatment of bacteriocins with surfactants may also eliminate some natural defenses of the indicator organisms. Finally, the observed anti-*Candida* activity of BLISs was not due to hydrogen peroxide, as activity was maintained after treatment with catalase.

Bacteriocins from streptococci have been recovered from *S. salivarius*, *S. mutans*, *S. pyogenes*, *S. bovis*, and *S. rattus* [10]. A nonpathogenic oral commensal bacterium, *S. salivarius* K12, exhibited antagonistic activity against oral *C. albicans* growth in vitro [51]. To our knowledge, this study is the first to report antagonistic activity of *S. uberis* against *Candida* isolates. Antibacterial bacteriocins from *S. uberis* (nisin U and uberolysin) have been biochemically characterized [52]. Although ubericin A was the first class IIa bacteriocin isolated from streptococci to be characterized [53], its activity against *Candida* has not been studied. The stronger bioactivity of ammonium-sulphate-purified products, compared to crude extracts, in all tested BLISs may be due to the increased concentration of the proteinaceous compounds [47].

Biofilm formation led to poor clinical outcomes with candidiasis [54]. In this study, BLISs from probiotic lactobacilli and streptococci induced a significant reduction in biofilm biomass. Previous studies showed that the biofilms of *C. albicans* were reduced by cells and supernatants of lactobacilli [19,20,55,56,57].

*L. acidophilus* ATCC 4356 was shown to protect *G. mellonella* against in vivo experimental candidiasis [55]. Similarly, probiotic bacteria (*L. rhamnosus* and *L. acidophilus*) significantly reduced *C. albicans* oral growth in immunocompromised mice [58]. In this study, we showed that injecting BLISs from *L. pentosus*, *L. delbrueckii* subsp. *lactis I*, and *L. paracasei* subsp. *paracasei II* into *G. mellonella* larvae infected with *Candida* strains significantly increased the survival of larvae. Another study showed that larvae receiving *L. rhamnosus* supernatants were more protected against *C. albicans* than larvae receiving *L. rhamnosus* cells [20]. This might be attributed to the immediate availability of bacteriocins or organic acids in the supernatant versus cells that still need to grow and produce antimicrobial bacteriocins [20].

The BLIS of *S. uberis II* significantly reduced the biofilm biomass of *Candida in vitro*. The in vivo protective activity significantly improved the larvae’s seventh-day survival (not the first- or the second-day survival) only when larvae were infected with *C. glabrata* clinical isolates, but not when infected with *C. albicans*. BLISs from *L. pentosus, L. paracasei* subsp. *paracasei II*, and *L. delbrueckii* subsp. *lactis I* had a significant protective action on larvae’s survival on all three testing days (1, 2, and 7) when larvae were infected with either *C. albicans* or *C. glabrata* clinical isolates. *L. rhamnosus* and *L. casei* have been previously reported to exhibit stronger antifungal activity than *S. thermophilus* and *S. salivarius* [59]. Parolin et al. showed that *C. albicans* isolates were more highly suppressed by lactobacilli than other *Candida* species [60].

Our in vitro and in vivo experiments showed that BLISs derived from potential probiotics have anti-*Candida* activity and can prevent biofilm formation. Furthermore, BLISs are tolerant to heat, nonproteolytic enzymes, pH, surfactants, and organic solvents. Thus, BLISs should be considered as an alternative or adjunct antimicrobial therapy to currently used antifungal agents for the treatment of VVC.

## Figures and Tables

**Table 1:** Inhibitory activity of different bacteriocin-like inhibitory substances (BLISs) on *Candida albicans* and non-*Candida albicans* isolates.

	*Candida albicans* Isolates (*n* = 25)		*Non-Candida albicans* Isolates (*n* = 20)		All *Candida* Isolates (*n* = 45)	*p* Value
Probiotic Isolates (as Sources of BLISs)	Positive Number (%)	Negative Number (%)	Positive Number (%)	Negative Number (%)	Positive Number (%)	
*Lactobacillus pentosus*	15 (60)	10 (40)	18 (90)	2 (10)	33 (73.3)	0.024 *
*Lactobacillus plantarum I*	12 (48)	13 (52)	13 (65)	7 (35)	25 (55.6)	0.254
*Streptococcus uberis I*	13 (52)	12 (48)	10 (50)	10 (50)	23 (51.1)	0.894
*Lactobacillus rhamnosus I*	17 (68)	8 (32)	13 (65)	7 (35)	30 (66.7)	0.832
*Lactobacillus delbrueckii* subsp. *bulgaricus*	13 (52)	12 (48)	14 (70)	6 (30)	27 (60)	0.221
*Lactobacillus paracasei* subsp. *paracasei I*	14 (56)	11 (44)	11 (55)	9 (45)	25 (55.6)	0.947
*Lactobacillus plantarum II*	13 (52)	12 (48)	13 (65)	7 (35)	26 (57.8)	0.380
*Lactobacillus paracasei* subsp. *paracasei II*	15 (60)	10 (40)	16 (80)	4 (20)	31 (68.9)	0.150
*Lactobacillus rhamnosus II*	14 (56)	11 (44)	12 (60)	8 (40)	26 (57.8)	0.787
*Lactobacillus delbrueckii* subsp. *lactis I*	17 (68)	8 (32)	13 (65)	7 (35)	30 (66.7)	0.832
*Lactobacillus delbrueckii* subsp. *lactis II*	15 (60)	10 (40)	14 (70)	6 (30)	29 (64.4)	0.486
*Streptococcus agalactiae*	17 (68)	8 (32)	12 (60)	8 (40)	29 (64.4)	0.577
*Streptococcus uberis II*	17 (68)	8 (32)	13 (65)	7 (35)	30 (66.7)	0.832

*, indicates statistical significance.

**Table 2:** Effect of heat, enzymes, and pH on bacteriocin-like inhibitory substances (BLISs).

Treatment			Probiotic Isolates (as Sources of BLISs)				
			*Lactobacillus* *pentosus*	*Lactobacillus* *rhamnosus I*	*Lactobacillus* *paracasei* subsp. *paracasei II*	*Lactobacillus* *delbrueckii* subsp. *lactis I*	*Streptococcus* *uberis II*
Effect of Heat	121 °C	15 min	+	+	+	−	−
	100 °C	30 min	+	+	+	+	−
		60 min	+	+	+	−	−
	80 °C	30 min	+	+	+	+	−
	40 °C	30 min	+	+	+	+	+
Effect of Enzymes	Pepsin		S	S	S	S	S
	Trypsin		S	S	S	S	S
	α-amylase		R	R	R	R	R
Effect of pH	pH 3		+	+	+	+	+
	pH 5		+	+	+	+	+
	pH 7		++	++	++	++	++
	pH 10		+	+	+	+	+

R: Resistant to inactivation; S: Sensitive to inactivation, +: Retained activity; ++: Retained enhanced activity, −: No activity.

**Table 3:** Effect of surfactants on bacteriocin-like inhibitory substances (BLISs) activity against *Candida albicans* and *Candida glabrata*, as indicated by residual activity (%).

Surfactant	Concentration	Probiotic Isolates (as Sources of BLISs)									
		*Lactobacillus* *pentosus*		*Lactobacillus rhamnosus I*		*Lactobacillus* *paracasei* subsp. *paracasei II*		*Lactobacillus* *delbrueckii* subsp. *lactis I*		*Streptococcus uberis II*	
		*Candida albicans*	*Candida glabrata*	*Candida albicans*	*Candida glabrata*	*Candida albicans*	*Candida glabrata*	*Candida albicans*	*Candida glabrata*	*Candida albicans*	*Candida glabrata*
Tween 20	0.1%	101.7%	70.7%	81.3%	96.4%	91.1%	79.3%	100.0%	88.6%	100.0%	75.3%
	1.0%	100.0%	70.7%	81.3%	94.9%	86.7%	77.9%	100.0%	75.9%	111.8%	78.2%
	2%	90.0%	67.3%	81.3%	91.4%	86.7%	80.6%	100.0%	75.9%	94.1%	102.9%
	5%	86.7%	70.7%	81.3%	91.4%	86.7%	74.9%	100.0%	63.3%	80.4%	69.9%
Tween 80	0.1%	100.0%	91.9%	75.0%	91.4%	113.3%	80.6%	95.8%	88.6%	100.0%	102.9%
	1.0%	100.0%	93.3%	75.0%	96.4%	106.7%	85.0%	91.7%	84.4%	105.9%	62.9%
	2%	90.0%	81.8%	68.8%	100.0%	106.7%	88.1%	95.8%	84.4%	100.0%	69.9%
	5%	90.0%	77.4%	68.8%	108.1%	106.7%	74.9%	95.8%	84.4%	94.1%	61.7%

**Table 4:** Biofilm-inhibitory activity of bacteriocin-like inhibitory substances (BLISs) of potential probiotics against *C. albicans* and *C. glabrata* clinical isolates and *C. albicans* ATCC 90028.

	*Candida albicans* Clinical Isolate (CA 1)			*Candida glabrata* Clinical Isolate (NCAC 1)			*Candida albicans* (ATCC 90028)		
Group	Mean ± SD	Percentage Reduction	*p*-Value	Mean ± SD	Percentage Reduction	*p*-Value	Mean ± SD	Percentage Reduction	*p*-Value
*No Lactobacilli*	6.182 ± 0.181	-	-	3.062 ± 0.110	-	-	21.744 ± 0.164	-	-
*Lactobacillus* *pentosus*	1.667 ± 0.055	73.0%	<0.0001 *	0.891 ± 0.055	70.9%	<0.0001 *	6.395 ± 0.055	70.6%	<0.0001 *
*Lactobacillus* *rhamnosus I*	2.733 ± 0.060	55.8%	<0.0001 *	1.403 ± 0.044	54.2%	<0.0001 *	10.977 ± 0.428	49.5%	<0.0001 *
*Lactobacillus paracasei* subsp. *paracasei II*	2.143 ± 0.071	65.3%	<0.0001 *	1.143 ± 0.082	62.7%	<0.0001 *	7.981 ± 0.115	63.3%	<0.0001 *
*Lactobacillus delbrueckii* subsp. *lactis I*	1.919 ± 0.027	69.0%	<0.0001 *	0.911 ± 0.005	70.3%	<0.0001 *	6.973 ± 0.115	67.9%	<0.0001 *
*Streptococcus uberis II*	3.671 ± 0.005	40.6%	<0.0001 *	1.450 ± 0.044	52.7%	<0.0001 *	12.682 ± 0.504	41.7%	<0.0001 *

*, indicates statistical significance.

**Table 5:** Survival of *Candida*-infected *Galleria mellonella* treated with bacteriocin-like inhibitory substances (BLISs) on the first, second, and seventh days.

	*Candida albicans* Clinical Isolate (CA 1)	First Day Survival		Second Day Survival		Seventh Day Survival	
		Number (%)	*p*-Value ^#^	Number (%)	*p*-Value ^#^	Number (%)	*p*-Value ^#^
	No BLIS (*Candida* only)	2 (12.5)	-	1 (6.3)	-	0 (0.0)	-
Probiotic Isolates (as sources of BLISs)	*Lactobacillus pentosus*	9 (56.3)	0.009 *	8 (50.0)	0.006 *	7 (43.8)	0.003 *
	*Lactobacillus rhamnosus I*	7 (43.8)	0.049	5 (31.3)	0.070	3 (18.8)	0.068
	*Lactobacillus paracasei* subsp. *paracasei II*	13 (81.3)	<0.0001 *	12 (75.0)	<0.0001 *	8 (50.0)	0.001 *
	*Lactobacillus delbrueckii* subsp. *lactis I*	12 (75.0)	<0.0001 *	10 (62.5)	<0.0001 *	8 (50.0)	0.001 *
	*Streptococcus uberis II*	5 (31.3)	0.199	4 (25.0)	0.146	1 (6.3)	0.308
	***Candida glabrata*** **clinical isolate (NCAC 1)**						
	No BLIS (*Candida* only)	3 (18.8)	-	2 (12.5)	-	0 (0.0)	-
Probiotic Isolates (as sources of BLISs)	*Lactobacillus pentosus*	13 (81.3)	<0.0001 *	13 (81.3)	<0.0001 *	8 (50.0)	0.001 *
	*Lactobacillus rhamnosus I*	9 (56.3)	0.029	7 (43.8)	0.049	4 (25.0)	0.033
	*Lactobacillus paracasei* subsp. *paracasei II*	12 (75.0)	0.001 *	11 (68.8)	0.001 *	9 (56.3)	<0.0001 *
	*Lactobacillus delbrueckii* subsp. *lactis I*	12 (75.0)	0.001 *	11 (68.8)	0.001 *	10 (62.5)	<0.0001 *
	*Streptococcus uberis II*	6 (37.5)	0.239	4 (37.5)	0.103	3 (35.4)	0.009 *
	***Candida albicans* ATCC 90028**						
	No BLIS (*Candida* only)	3 (18.8)	-	3 (18.8)	-	0 (0.0)	-
Probiotic Isolates (as sources of BLISs)	*Lactobacillus pentosus*	9 (56.3)	0.029	8 (50.0)	0.063	6 (37.5)	0.007 *
	*Lactobacillus rhamnosus I*	6 (37.5)	0.239	5 (31.3)	0.415	4 (25.0)	0.033
	*Lactobacillus paracasei* subsp. *paracasei II*	11 (68.8)	0.004 *	11 (68.8)	0.004 *	9 (56.3)	<0.0001 *
	*Lactobacillus delbrueckii* subsp. *lactis I*	12 (75.0)	0.001 *	12 (75.0)	0.001 *	8 (50.0)	0.001 *
	*Streptococcus uberis II*	5 (31.3)	0.415	4 (25.0)	0.672	2 (12.5)	0.144

^#^, chi-squared test; *, indicates statistical significance at *p* < 0.01.

## Data Availability

Not applicable.
